# A novel multion in a Chinese family with neurofibromatosis type 1: A case report

**DOI:** 10.1097/MD.0000000000045455

**Published:** 2025-10-31

**Authors:** Xiaoran Tao, Xiaoli Yang, Xinyu Huang, Min Fan, Zaixing Wang

**Affiliations:** a Department of Dermatology, The First Affiliated Hospital, Anhui Medical University, Hefei, Anhui China; b Institute of Dermatology, Anhui Medical University, Hefei, Anhui, China.

**Keywords:** case report, family, mutation, neurofibromatosis type 1

## Abstract

**Rationale:**

Neurofibromatosis type 1 (NF1), an autosomal dominant genetic disorder, exhibits a high prevalence across populations. The quintessential clinical manifestations of NF1 encompass a spectrum of features, including neurofibromas, café-au-lait macules (CALMs), skinfold freckling, Lisch nodules, and an array of central nervous system tumors. The pathogenesis of NF1 is intricately tied to mutations within the *NF1* gene, situated on chromosome 17q11.2. This gene encodes the neurofibromin protein, whose functional loss leads to deregulated cell growth, thereby fostering an environment conducive to tumorigenesis.

**Patient concerns:**

A 23-year-old female developed freckles under her armpits more than a decade ago. CALMs and hypertrophic papules appeared on her trunk and extremities. Their size and quantity gradually increased, with the largest lesion reaching 3.1 cm. There were no specific discomforts such as itching or pain.Her sister and mother have the same manifestations.

**Diagnoses:**

The proband has more than 6 CALMs and more than 2 neurofibromas visible all over the body, with scattered freckles in the axillae. Similar rashes are also observed in his/her mother and younger sister. Therefore, the patient is diagnosed with NF1.

**Interventions:**

As the proband has a need for pregnancy, we performed Sanger sequencing on the genes of the proband and their family members.

**Outcomes:**

A novel mutation located in the *NF1* gene was identified, and genetic counseling was provided to the proband.

**Lessons:**

This study unveiled a novel pathogenic nonsense mutation, designated as *NF1* c.240_243del (p.Q83*), within the proband’s genetic sequence. This mutation introduces a premature stop codon, resulting in the truncation of the neurofibromin protein. This truncation, in turn, precipitates the onset of NF1, underscoring the critical role of the *NF1* gene in maintaining normal cellular growth and proliferation.

## 1. Introduction

Neurofibromatosis type 1 (NF1) stands as a prevalent autosomal dominant genetic disorder, estimated to affect approximately 1 in every 3000 individuals worldwide. The clinical tapestry of NF1 is woven with characteristic manifestations such as neurofibromas, café-au-lait macules (CALMs), skinfold freckling, Lisch nodules, and an array of central nervous system tumors.^[1]^ The roots of NF1 pathogenesis are firmly anchored in mutations within the *NF1* gene, situated on chromosome 17q11.2, which encodes the vital protein neurofibromin. The resultant loss of neurofibromin function disrupts cellular growth regulation, fostering an environment conducive to tumorigenesis.^[2]^

## 2. Case report

**Figure 1.**
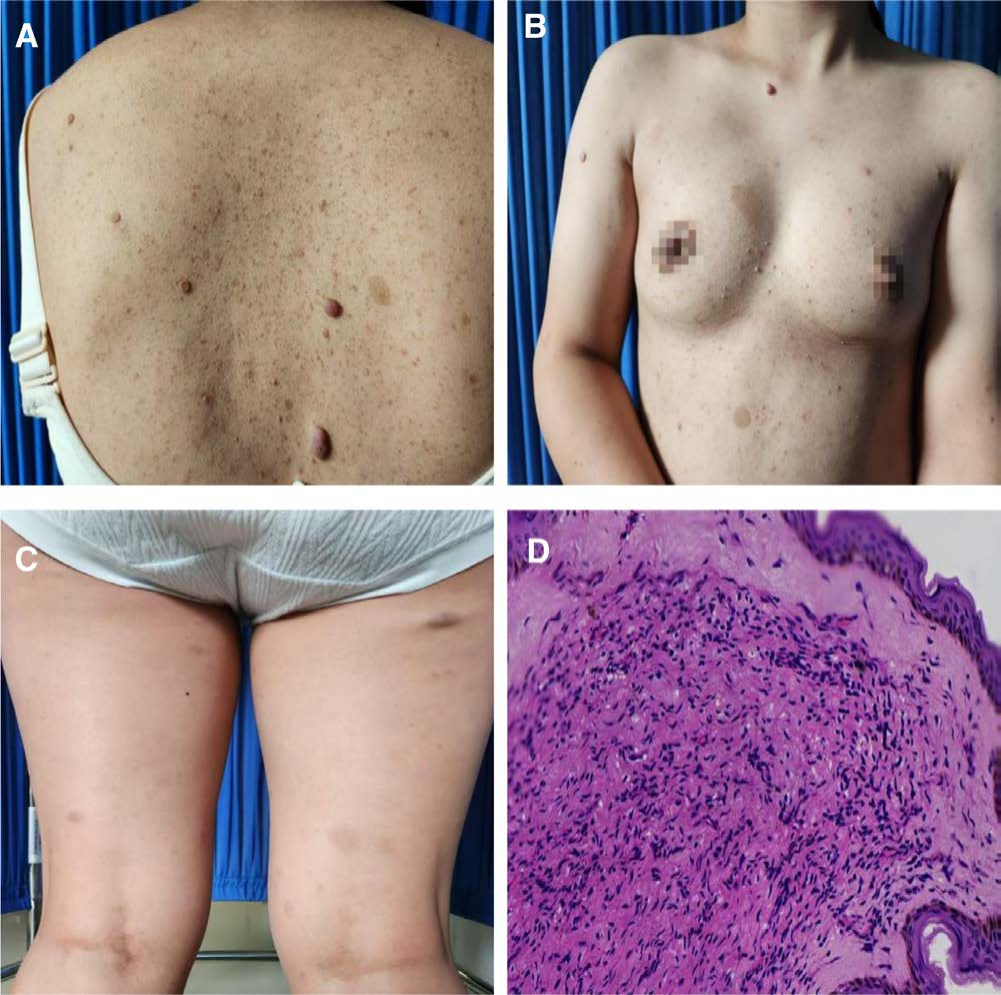
(A and B) CALMs, freckles, and soft papules observed on the proband’s chest, abdomen, and back. (C) A light bluish subcutaneous nodule on the proband’s right lower limb. (D) Histopathology of the subcutaneous mass in the proband (H&E, ×40), showing thinning of the epidermis and clusters of spindle-shaped cells in the dermis, with the spindle cells exhibiting tapered ends. CALMs = café-au-lait macules.

The proband, a 23-year-old female, first exhibited scattered CALMs of varying sizes across her trunk just 1 month after birth. These lesions progressively multiplied and expanded in size over time. By the age of 12, she noticed subcutaneous nodules ranging from 0.3 to 3.1 cm in the lumbar and abdominal regions, which subsequently increased in number and spread to her extremities and axillary areas (Fig. 1A–C), she has never taken it seriously. Now, in order to get pregnant, the proband comes for a consultation. For diagnostic clarification, a nodule was excised for histopathological examination, revealing epidermal thinning and clusters of spindle-shaped cells in the dermis. These spindle cells, characterized by their tapered ends, were consistent with a neurofibroma diagnosis (Fig. 1D).

**Figure 2.**
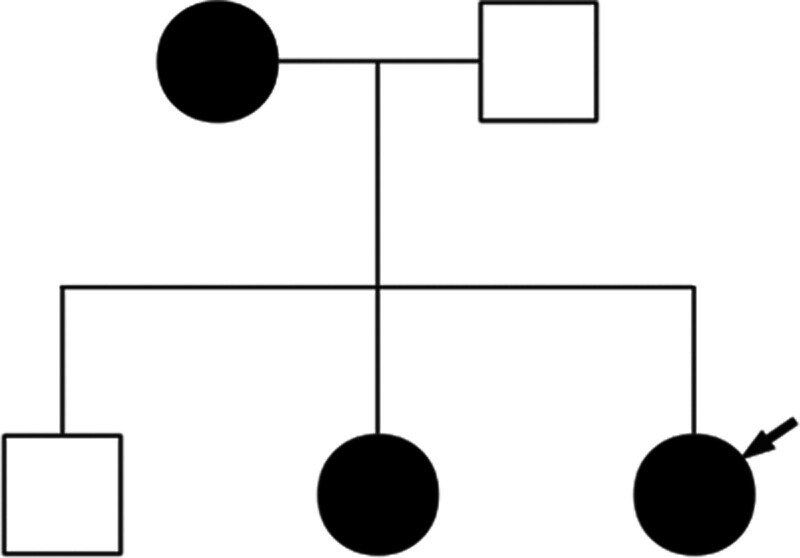
Pedigree of the family with NF1. Squares represent males, and circles represent females. The arrow indicates the proband. NFI = neurofibromatosis type 1.

**Figure 3.**
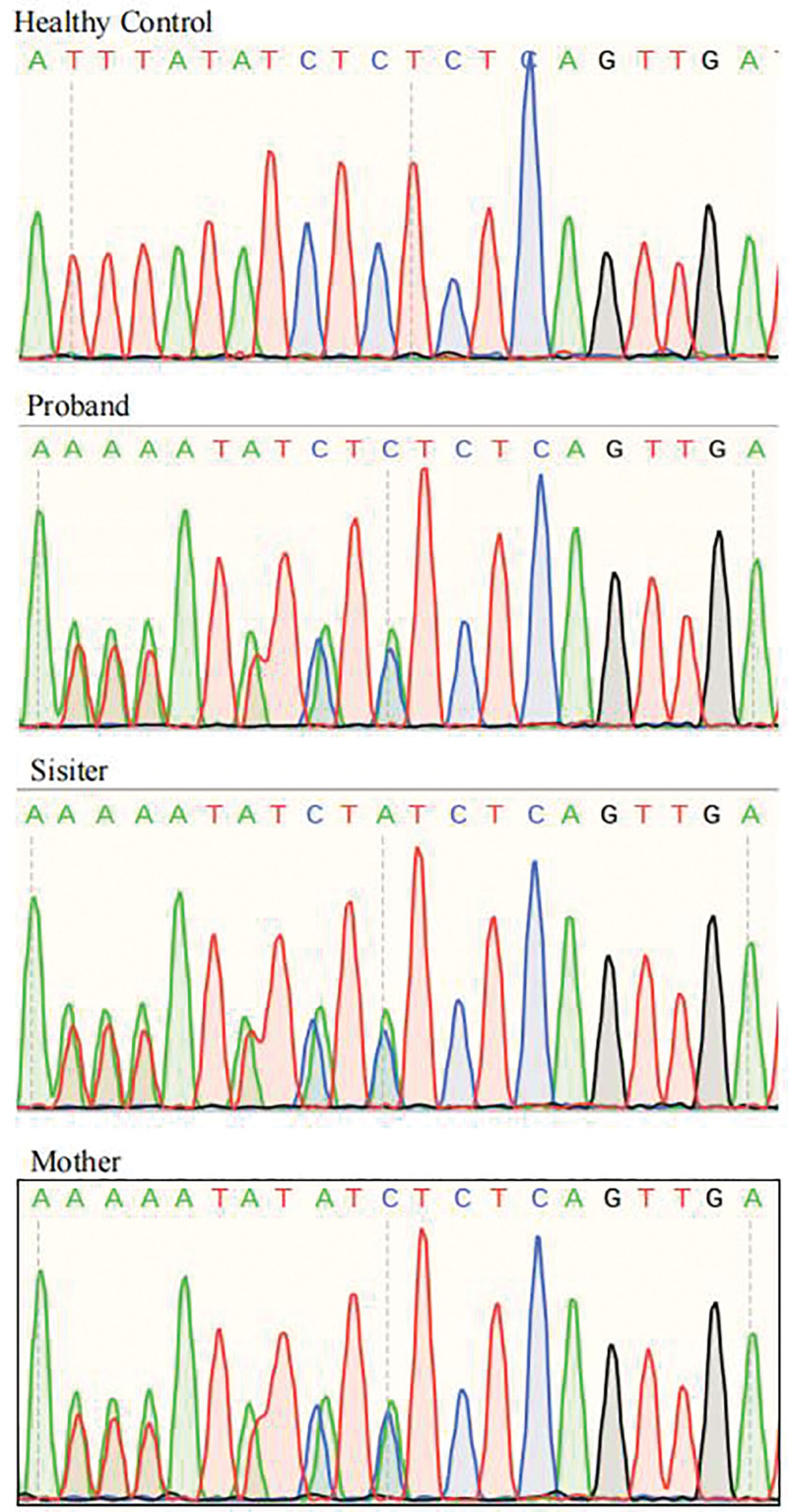
Sanger sequencing of the healthy control and the proband’s family revealed a novel frameshift mutation, c.240_243del, in exon 3 of the *NF1* gene in the proband, her sister, and mother, while no abnormalities were observed in the healthy control. NFI = neurofibromatosis type 1.

The proband’s mother, a 45-year-old female, presented with lesions primarily confined to the lumbar and abdominal regions, encompassing CALMs, freckling, and scattered subcutaneous nodules. The proband’s 17-year-old sister exhibited milder skin manifestations, with scattered subcutaneous nodules and freckles limited to both upper limbs. Conversely, the proband’s father and younger brother displayed no pertinent clinical signs or symptoms (Fig. 2).In an act of voluntary participation, the proband and her family members affixed their signatures to the informed consent forms, consenting to the procedures outlined. Subsequently, samples of their peripheral blood were meticulously collected for the purpose of whole-exome sequencing, with Sanger sequencing serving as a rigorous validation step. The resultant analysis unveiled a mutation within exon 4 of the *NF1* gene – specifically, a deletion (c.240_243del) leading to a premature stop codon (p.Q83*) – in the proband, her mother, and her sister (Fig. 3). This frameshift mutation introduces an abrupt termination at amino acid position 83, producing a truncated protein comprising merely 83 amino acids, thereby severely compromising its functional integrity.

We informed the proband of relevant treatment options, such as surgical resection and the use of the small - molecule MEK inhibitor (Selumetinib). She stated that she would not consider receiving treatment for the time being. As the proband currently has a need for pregnancy, we provided her with detailed genetic counseling, and the patient expressed satisfaction.

The c.240_243del mutation identified within this family is a novel entity, unrecorded in the esteemed Human Gene Mutation Database or the ClinVar databases. In accordance with the stringent guidelines formulated by the American College of Medical Genetics,^[3]^ this mutation is categorically classified as pathogenic, based on a constellation of criteria (PSV1 + PM2 + PM4 + PP2 + PP4).

## 3. Discussion

NF1 is a complex, multisystem genetic disorder characterized by a spectrum of mutations within the *NF1* gene. These mutations encompass nonsense mutations, frameshift mutations, splice site mutations, and missense mutations, each with the potential to abolish the function of neurofibromin. The resultant disruption in the normal regulation of the Ras signaling pathway fosters an environment conducive to cell proliferation and tumor formation.^[4]^

A previous study highlighted the case of siblings with NF1 who harbored an identical genetic mutation (c.5392C > T, p.Gln1798Ter). While the male sibling exhibited a limited number of cutaneous and subcutaneous manifestations (CALMs and nodules), the female sibling additionally presented with plexiform neurofibromas.^[5]^ Similarly, in the present report, the phenotypic disparities between the proband and her sister are striking. Their skin pigmentation patterns differ markedly, and the distribution of subcutaneous nodules varies considerably. The underlying causes of these phenotypic variations may be attributed to a interplay of genetic modifiers and environmental factors, underscoring the intricate interplay that governs the manifestation of *NF1*. However, this study is not devoid of certain limitations. Notably, the inability to establish contact with the proband’s mother and other relatives has resulted in a sample size that falls short of ideal. Furthermore, the absence of functional testing for the mutated gene represents another gap in our research endeavors.

## 4. Conclusion

This study has made a significant contribution by identifying a novel nonsense mutation, c.240_243del (p.Q83*), within the *NF1* gene. This discovery offers fresh insights into the ever-expanding mutation spectrum of the *NF1* gene, thereby deepening our understanding of its pathogenesis. It also holds promise for more refined genetic counseling and improved disease management strategies in clinical practice.

## Acknowledgments

We extend our heartfelt gratitude to the proband and her family for their invaluable support and willingness to participate in this study.

## Author contributions

**Conceptualization:** Xiaoran Tao, Zaixing Wang.

**Data curation:** Xiaoran Tao, Xiaoli Yang.

**Investigation:** Min Fan, Zaixing Wang.

**Supervision:** Zaixing Wang.

**Validation:** Zaixing Wang.

**Writing – original draft:** Xiaoran Tao, Xinyu Huang.

**Writing – review & editing:** Xiaoran Tao, Xiaoli Yang, Min Fan, Zaixing Wang.
